# DNA damage response pathway alterations in urothelial carcinoma: a road to precision oncology or a dead-end street?

**DOI:** 10.1093/oncolo/oyag284

**Published:** 2026-07-23

**Authors:** Patricia Guerrero Serrano, Pilar Sotoca, Adriana García, Juan Carlos Calvo, Carlos González-Merino, Coral García de Quevedo, José Daniel Subiela, Carolina Bueno, Víctor Albarrán, Aurea Molina, Begoña Pérez-Valderrama, Pablo Gajate

**Affiliations:** Department of Medical Oncology, Ramón y Cajal University Hospital, M-607, Km. 9, 100, Fuencarral-El Pardo, Madrid 28034, Spain; Department of Medical Oncology, Ramón y Cajal University Hospital, M-607, Km. 9, 100, Fuencarral-El Pardo, Madrid 28034, Spain; Department of Medical Oncology, Ramón y Cajal University Hospital, M-607, Km. 9, 100, Fuencarral-El Pardo, Madrid 28034, Spain; Department of Medical Oncology, Ramón y Cajal University Hospital, M-607, Km. 9, 100, Fuencarral-El Pardo, Madrid 28034, Spain; Department of Medical Oncology, Ramón y Cajal University Hospital, M-607, Km. 9, 100, Fuencarral-El Pardo, Madrid 28034, Spain; Department of Medical Oncology, Ramón y Cajal University Hospital, M-607, Km. 9, 100, Fuencarral-El Pardo, Madrid 28034, Spain; Department of Urology, Ramón y Cajal University Hospital, M-607, Km. 9, 100, Fuencarral-El Pardo, Madrid 28034, Spain; Department of Urology, Infanta Sofía Hospital, Paseo Europa, 34, San Sebastián de los Reyes, Madrid 28702, Spain; Translational Genomics and Targeted Therapies Group, Cell Therapy Unit, Clinic Barcelona Comprehensive Cancer Center, IDIBAPS, Carrer de Villarroel, 170, Barcelona 08036, Spain; Department of Medical Oncology, Complejo Hospitalario Universitario de La Coruña, Xubias de Arriba, 84, La Coruña 15006, Spain; Department of Medical Oncology, Hospital Universitario Virgen del Rocío, Avenida, Manuel, Siurot S/N, Seville 41013, Spain; Department of Medical Oncology, Ramón y Cajal University Hospital, M-607, Km. 9, 100, Fuencarral-El Pardo, Madrid 28034, Spain

**Keywords:** urothelial carcinoma, DNA damage response, DDR alterations, platinum chemotherapy, immune checkpoint inhibitors, PARP inhibitors

## Abstract

Urothelial carcinoma ranks among the most common solid tumors and exhibits aggressive behavior, with limited survival in the metastatic setting despite recent therapeutic advances. Currently, 3 first-line systemic treatment options are supported by level IA evidence, yet no validated predictive biomarkers exist to guide selection among them. Alterations in DNA damage response (DDR) pathways occur in a substantial proportion of urothelial tumors and have emerged as potential predictive biomarkers of treatment sensitivity. This review examines the biological basis of DDR pathways and their implications in carcinogenesis, summarizes the frequency and spectrum of DDR gene alterations in urothelial carcinoma, and critically appraises the available evidence linking these alterations to responses to platinum-based chemotherapy, immune checkpoint inhibitors, and PARP inhibitors in both muscle-invasive and metastatic settings. Although retrospective data suggest associations between DDR alterations and improved outcomes with certain therapies, results across studies are heterogeneous, likely reflecting inconsistent definitions of DDR alterations, the variable functional impact of individual mutations, and differences in patient populations. We discuss these limitations and highlight the need for standardized criteria and prospective validation to determine whether DDR pathway alterations can be reliably integrated into clinical decision-making for patients with urothelial carcinoma.

Implications for Practice DDR pathway alterations are emerging as promising candidate biomarkers in urothelial carcinoma. Retrospective evidence suggests that these alterations may identify patients with increased sensitivity to platinum-based chemotherapy, immune checkpoint inhibitors, and PARP inhibitors. However, most studies lack the comparator groups and biomarker-treatment interaction analyses required to establish treatment-specific predictive value. In addition, heterogeneous definitions of DDR alterations and the variable functional impact of individual mutations currently limit their clinical applicability. Standardization of molecular criteria and prospective validation are needed before DDR biomarkers can be routinely integrated into therapeutic decision-making. Nevertheless, DDR profiling may help refine treatment sequencing and support the development of personalized therapeutic strategies in urothelial carcinoma.

## Introduction

Urothelial carcinoma ranks among the 10 most common solid tumors and exhibits aggressive behavior. Approximately 10% of cases present with distant metastases at diagnosis, and in non-metastatic muscle-invasive disease, relapse rates remain high despite radical-intent treatment (20%-30% at 5 years in N0 patients and up to 60%-75% in the presence of lymph node involvement).^1^^,^^2^ For decades, platinum-based chemotherapy (CT) has been the standard treatment for metastatic urothelial carcinoma (mUC), with very limited outcomes in terms of overall survival (OS), around 10 to 14 months.^3^^,^^4^ To address this unmet need, new drugs have been developed that expand the therapeutic arsenal and significantly improve survival. Currently, there are 3 first-line systemic treatment options, all with a level IA evidence:^5^ platinum-based CT followed by maintenance avelumab according to the JAVELIN Bladder-100 study;^6^ enfortumab vedotin (EV) + pembrolizumab (P) based on EV-302;^7^ and cisplatin + gemcitabine + nivolumab according to the CheckMate 901 results.^8^ Although the combination of EV + P is recommended as the preferred option across several clinical guidelines, the availability of multiple alternative treatments supported by comparable levels of evidence raises the question of how to select the therapy most likely to provide the greatest benefit for an individual patient. In this context, predictive factors of response are of increasing importance, as they can guide the selection among therapeutic options.

DNA damage response (DDR) pathway alterations have been associated with outcomes following platinum-based chemotherapy (CT), immune checkpoint inhibitors (ICI), and poly(ADP-ribose) polymerase inhibitors (PARPi). Emerging preclinical evidence also suggests that DNA repair deficiency may influence sensitivity to antibody-drug conjugate payloads, although its clinical relevance remains unclear. As most available studies are retrospective, single-arm, or lack biomarker-treatment interaction analyses, these associations do not establish treatment-specific predictive value. DDR alterations may nevertheless help identify therapeutic vulnerabilities and refine treatment sequencing. This review examines their association with treatment outcomes and their potential future role in therapeutic decision-making.

## Materials and methods

A non-systematic narrative literature review was conducted using PubMed/MEDLINE, Embase, and ClinicalTrials.gov databases. Relevant studies evaluating DNA damage response (DDR) pathway alterations in urothelial carcinoma and their association with response to platinum-based chemotherapy, immune checkpoint inhibitors, and PARP inhibitors were identified. Priority was given to prospective clinical trials, retrospective translational studies, and high-impact reviews published in English. Abstracts from major oncology meetings, including ASCO and ESMO, were also reviewed when considered clinically relevant. Articles were selected based on their relevance to the biological rationale, prognostic implications, and potential treatment-associated relevance of DDR alterations in urothelial carcinoma. This review was not conducted according to a systematic review protocol, and no PRISMA-based study selection process was applied.

## Results

### DDR pathways: implications in carcinogenesis and sensitivity to anticancer treatment

Genomic integrity is threatened by endogenous replication stress and external insults, requiring a coordinated network of DDR pathways to detect and repair DNA lesions. These mechanisms are essential to maintain genomic stability through precise cell-cycle control and accurate repair processes. When DDR pathways are altered, cells progressively accumulate mutations and chromosomal abnormalities that promote malignant transformation. Accordingly, DDR dysfunction is now recognized as a central driver of carcinogenesis and a key source of therapeutic vulnerability in cancer. To maintain genomic integrity, healthy cells rely on 4 main pathways to repair DNA damage.

#### Base excision repair

Corrects single-base DNA alterations caused by oxidation, deamination, or alkylation.^9^ Alterations in this pathway increase the risk of developing neoplasms, especially in tissues exposed to oxidative stress or external carcinogens such as the lung, head and neck, or colon.^10^ Tumors with BER alterations show increased susceptibility to alkylating agents, which induce DNA damage that cannot be repaired.^11–14^

#### Nucleotide excision repair

Removes bulky lesions that distort the DNA helix, such as pyrimidine dimers induced by ultraviolet radiation.^15^ NER deficiencies are associated with skin and ocular tumors in sun-exposed areas.^16^^,^^17^ Alterations in this pathway increase sensitivity to alkylating agents and platinum compounds, which act by distorting the DNA double helix.^18–20^

#### Mismatch repair

Corrects errors that occur during DNA replication.^21^ Deficiency in this pathway (dMMR) due to mutations in genes such as MLH1, MSH2, MSH6, and PMS2 generates microsatellite instability (MSI), resulting in tumors with a higher tumor mutational burden (TMB).^22^ Consequently, there is increased neoantigen expression, enhancing tumor immunogenicity. For this reason, ICI treatment is more effective in this context.^23–25^

#### Double-strand break repair

Double-strand DNA breaks are repaired through 2 mechanisms:

- Non-Homologous End Joining (NHEJ): Directly ligates fragmented DNA ends without the need for a homologous template. This pathway predominates in the G1 phase and is the fastest repair mechanism but prone to errors.^26–28^
- Homologous Recombination (HR): Uses a homologous sequence as a template to repair DNA breaks accurately. It predominates in the S and G2 phases of the cell cycle.^26^^,^^27^^,^^29^

Tumors with homologous recombination deficiency (HRD) rely on PARP activity to repair DNA breaks via alternative pathways such as NHEJ, explaining their increased sensitivity to PARPi.^30^^,^^31^ Additionally, in this context, a synergistic effect between PARPi and ICI has been proposed, as PARP inhibition induces tumor cell death, exposing DNA fragments that activate the cGAS-STING pathway and lead to PD-L1 overexpression, enhancing the antitumor immune response.^32–34^

Key genes involved in each DNA damage response (DDR) pathway are summarized in Table 1.

**Table 1.** DNA damage response pathways and representative genes.

Pathways to repair DNA damage		Relevant genes
**Base excision repair (BER)**		APE1, POLB, XRCC1, LIG3, PARP1/2, MUTYH and genes encoding DNA glycosylases^14^
**Nucleotide excision repair (NER)**		ERCC1, ERCC2, ERCC3, ERCC4, ERCC5, XPA, XPC, DDB2, CSA, CSB, and RAD23B^20^
**Mismatch repair (MMR)**		MLH1, MSH2, MSH6, and PMS2^22^
**Double-strand break repair (DSB)**	**NHEJ**	XRCC6, XRCC5, PRKDC, DCLRE1C, POLM, POLL, LIG4, XRCC4, NHEJ1, PAXX, MRI/CYREN, TDP-43, TP53BP1 ERCC6L2, and RNase H2^28^
	**HR**	BRCA1, BRCA2, PALB2, RAD51, RAD51B, RAD51C, RAD51D, XRCC2, XRCC3, ATM, ATR, CHEK2, BARD1, BRIP1, MRE11A, NBN, RAD50, RPA1, DMC1, BLM, TOP3A, TOPBP1, RIF1, RMI1, and RMI2^29^

### DDR pathways in mUC

The frequency of DDR gene alterations in urothelial carcinoma is variable: while some sequencing studies have detected alterations in up to 45% of cases,^35^ others report frequencies of around 10%-25%.^36–38^ These differences are due to the lack of uniform criteria for defining what constitutes a DDR alteration. In the first study, alterations included variants that were not always pathogenic, such as silent mutations or variants of uncertain significance, whereas other studies selected only mutations considered deleterious. The specific mutation frequency of each gene is heterogeneous across cohorts, although ATM, ERCC2, BRCA1/2, PRKDC, and ATRX have been reported as the most frequently mutated genes in urothelial tumors.^36^^,^^37^^,^^39^ The summarized mutation frequencies for these genes are presented in Table 2.

**Table 2.** Frequency of genetic alterations in DNA damage response pathways in urothelial tumors.^36^^,^^37^^,^^39^

Gene	DDR pathway	Frequency in urothelial tumors (%)
**ATM**	Homologous recombination	5-13
**ERCC2**	Nucleotide excision repair	7-12
**BRCA1/2**	Homologous recombination	1-4
**PRKDC**	Non-homologous end joining	1-3
**BRIP1**	Homologous recombination	1-2
**PALB2**	Homologous Recombination	1-2
**CHEK2**	Homologous recombination	1-2
**ATR**	Homologous recombination	<1-2
**RAD50**	Homologous recombination	<1-2

Alterations in DDR have been associated with improved progression free survival (PFS) and overall survival (OS)^40^ and have shown a trend toward a lower prevalence of visceral involvement,^41^ suggesting a better prognosis in this subgroup of patients.

### Systemic treatment in mUC with DDR pathway alterations

#### Chemotherapy

The inability of tumors with DDR alterations to repair DNA damage explains their increased susceptibility to platinum-based agents. This relationship has been supported by studies conducted in both the neoadjuvant and metastatic settings.

In the study by Teo et al, mUC patients treated with platinum-based CT were stratified according to the presence or absence of alterations in a panel of 34 DDR genes (47% of the cohort harbored DDR alterations). This subgroup showed improved PFS (9.3 vs 6.0 months, *P* = .007) and OS (23.7 vs 13.0 months, *P* = .006).^51^ Another retrospective analysis of 3 independent cohorts of mUC patients treated with platinum showed that DDR gene alterations, excluding ATM, were associated with better survival (HR, 0.49; *P* = .003). However, ATM alterations correlated with poorer response to platinum-based treatment (HR, 2.03; *P* = .04).^40^ Other studies have reported worse survival in this patient subgroup regardless of treatment received, indicating that ATM alteration is an independent adverse prognostic marker in mUC patients.^42^^,^^43^ These findings support an association between DDR alterations and favorable outcomes following platinum-based CT, although their treatment-specific predictive value remains uncertain.

In the setting of non-metastatic muscle-invasive bladder cancer (MIBC), clinical outcomes have been retrospectively evaluated based on DDR alterations. A phase II cohort study showed that among patients treated with platinum-based neoadjuvant CT, the subgroup with mutations in ATM, RB1, or FANCC had significantly higher 5-year OS and disease-specific survival (DSS) rates: 85% vs 46% (*P* = .0043) and 90% vs 49% (*P* = .0015), respectively.^44^ These findings were later validated in a larger multicenter cohort (S1314), in which ERCC2 alterations were also associated with complete pathological response (pT0). In this validation, mutations showed a higher negative predictive value (86%) than positive predictive value (48%), indicating that patients without mutations in ATM, RB1, FANCC, or ERCC2 are less likely to achieve a complete pathological response after neoadjuvant CT.^45^ These predictive values reflect the ability of the biomarker panel to discriminate pathological response and should not be interpreted as evidence of a treatment-specific predictive effect.

The study by Van Allen et al analyzed tumor and germline DNA from 50 MIBC patients before receiving platinum-based neoadjuvant CT followed by radical cystectomy and observed that patients with complete response (pT0/pTis) had a higher frequency of somatic ERCC2 mutations compared to non-responders. This finding supports the hypothesis that alterations in the NER pathway, specifically in ERCC2, increase platinum sensitivity and may help select candidates for neoadjuvant CT.^46^ The association between ERCC2 mutations and pathological response to cisplatin-based neoadjuvant CT was independently replicated by Liu et al,^47^ supporting the robustness of this treatment-associated biomarker signal.

The association between DDR gene alterations and complete pathological response after neoadjuvant CT has motivated combining these parameters to select candidates for bladder preservation. The RETAIN-1 study is a single-arm phase II trial that included MIBC patients (cT2-T3N0M0). All patients underwent sequencing for mutations in ATM, ERCC2, FANCC, and RB1. Patients received neoadjuvant CT and a second TURBT post-CT. Those with at least 1 mutation in the analyzed genes and achieving cT0 after neoadjuvant therapy entered active surveillance instead of cystectomy. In the active surveillance group, 2-year metastasis-free survival was 76% (95% CI, 54.2-88.4) vs 71.1% (95% CI, 55.5-82.1) in the rest, although the pre-specified non-inferiority criterion was not met. In the active surveillance group, 48% maintained an intact bladder without developing metastases, and only 11% developed distant metastases. However, the local recurrence rate was high (∼68%), so while this strategy appears promising for bladder preservation in selected patients, it requires close monitoring and confirmation in larger series.^48^

The predictive value of DDR alterations for response to platinum-based CT in mUC patients is being prospectively evaluated in the SELECTIO-UC study, conducted by Roberto Iacovelli, with results still pending.^49^

#### ICI

Furthermore, somatic mutations in DDR pathways can lead to genomic instability and increased tumor mutational burden (TMB), which may enhance neoantigen presentation and improve immune recognition. Indeed, several retrospective studies in urothelial carcinoma have shown that alterations in DDR genes are associated with an elevated TMB^41^^,^^43^ and an increased tumor-infiltrating lymphocytes.^50^

In line with this, DDR gene alterations have also been associated with improved responses to ICIs. Joshi et al evaluated the correlation between alteration in DDR genes and response to anti-PD-1/PD-L1 therapies. DDR alterations (including ATM) were associated with a non-significantly higher ORR to PD-1/PD-L1 blockade (41% vs 21%, *P* = .136).^43^ Another study of 60 advanced urothelial patients treated with ICI observed that 47% patients had DDR alterations and 25% had known or likely deleterious DDR mutations. The presence of DDR alterations was correlated with best objective response, showing that the presence of any DDR alteration was associated with a higher response rate (67.9% vs 18.8%; *P* < .001). This association was more pronounced in patients whose tumors harbored known or likely deleterious DDR alterations (80%) than in those with DDR alterations of unknown significance (54%) or wild-type DDR genes (19%; *P* < .001).^36^

Finally, in the phase II PURE-01 trial evaluating neoadjuvant pembrolizumab in patients with MIBC, 52% of patients harbored deleterious DDR and/or RB1 gene alterations. DDR gene alterations were associated with higher pT0 response rates at cystectomy.^51^

RETAIN-2 met its primary endpoint, supporting the feasibility of response-adapted bladder preservation after neoadjuvant accelerated MVAC plus nivolumab in selected DDR-altered tumors, although prospective validation remains necessary.^52^

Overall, these studies support an association between DDR alterations and favorable outcomes following ICI, although the absence of non-ICI comparator groups precludes determining whether DDR alterations are specifically predictive of ICI benefit or reflect favorable tumor biology.

#### PARPi

PARP inhibitors impair single-strand DNA break repair and promote PARP trapping, leading to replication fork collapse and double-strand breaks. Tumors with impaired homologous recombination are less able to repair this damage and may therefore be sensitive to PARP inhibition through synthetic lethality.^30^^,^^31^

Given that a significant proportion of urothelial tumors harbor DDR alterations, PARPi have been investigated as a potential therapeutic strategy. Their use has been studied both in maintenance settings after response to CT and in combination with ICI in treatment-naïve patients.

The Atlantis study randomized patients with mUC and DDR alterations who had not progressed on platinum-based CT to receive maintenance rucaparib vs placebo. A clinical benefit in terms of PFS was observed in favor of rucaparib (35.3 weeks vs 15.1 weeks, HR, 0.53, *P* = .07).^53^ However, the Meet-URO12 study, with a similar design (mUC patients who had not progressed on CT), failed to demonstrate superiority of niraparib over placebo, even in the subgroup of patients with HR pathway mutations.^54^

Rucaparib also showed no efficacy in pretreated patients: the ATLAS study was a single-arm trial in which patients who had progressed after 1-2 lines of systemic therapy received full-dose rucaparib. PFS was 1.8 months, and no confirmed objective responses were observed, regardless of HR status.^55^ Olaparib monotherapy has similarly shown very limited activity in pretreated or platinum-ineligible mUC patients with DDR alterations, with no objective responses and PFS under 2 months.^56^ Response data have been reported in urothelial tumors with BRCA1/2 mutations,^57^ consistent with results observed in other solid tumors such as prostate and ovarian cancer.^58^^,^^59^

The discordant results of PARPi monotherapy in urothelial tumors have prompted exploration of combination strategies. The Bayou study evaluated the synergy of PARPi with ICI. Platinum-ineligible, treatment-naïve mUC patients were randomized to durvalumab + olaparib vs durvalumab + placebo. While the intention-to-treat population analysis did not show statistically significant differences, a subgroup analysis revealed improved PFS in patients with HRD in favor of the combination.^60^ However, in pretreated patients, the BISCAY study did not report benefits for durvalumab + PARPi vs durvalumab monotherapy, regardless of HR status.^61^ The combination of durvalumab + olaparib has also been explored as neoadjuvant therapy in MIBC. The NEODURVARIB study reported response rates higher than those achieved with CT (pathologic complete response in 44.5%), although no association with DDR status was observed.^62^

A systematic review by Crabb et al, analyzing multiple clinical trials of PARPi in mUC, supports the hypothesis that these drugs do not demonstrate efficacy in the general population but show promising results in patients with DDR alterations, particularly in the HRD subgroup.^63^ These findings suggest that future evaluations of PARPi in mUC should focus on patients selected according to molecular alterations in DDR pathways.

## Discussion

In both the MIBC and mUC settings, the incorporation of multiple therapeutic alternatives has highlighted the need for predictive biomarkers of response to optimize treatment selection.

In the mUC setting, current scientific evidence supports 3 first-line therapeutic options, all with level IA evidence,^6–8^ which adds complexity to initial treatment selection and subsequent sequencing. This underscores the need for validated predictive biomarkers to optimize therapeutic approaches for each patient. To date, most attempts to establish predictive biomarkers in urothelial tumors have been unsatisfactory,^64^ and only FGFR and PD-L1 have been validated for therapy selection.^65^^,^^66^

Alterations in genes involved in DDR pathways can be considered a double-edged sword: on one hand, they promote carcinogenesis, while on the other, they may confer increased sensitivity to certain treatments. Retrospective studies have associated these alterations with higher TMB and a lower incidence of visceral metastases, which in turn correlates with better prognosis.^41^ A central challenge is distinguishing prognostic from predictive effects. Prognostic biomarkers are associated with outcomes irrespective of treatment, whereas predictive biomarkers identify differential benefit from a specific therapy and generally require demonstration of a biomarker-treatment interaction in an adequately controlled study. Most DDR studies in urothelial carcinoma are retrospective or single-arm and cannot fully distinguish treatment-specific sensitivity from baseline tumor biology. Nevertheless, favorable outcomes across several treatment settings provide a rationale for investigating DDR alterations as candidate predictive biomarkers. These hypothesis-generating signals require prospective validation in appropriately designed studies. The methodological characteristics and level of inference supported by the key studies are summarized in Table 3.

**Table 3.** Key clinical studies evaluating the prognostic and treatment-associated relevance of DDR alterations in urothelial carcinom.

Study	Setting	Treatment	Design/phase	DDR biomarker	Outcomes	Inference supported
**Platinum-based chemotherapy**						
**Teo et al.^41^**	mUC	Platinum-based CT	Retrospective cohort	34-gene DDR panel	Longer PFS and OS with DDR alterations	Treatment-associated association; predictive value not established
**Yin et al.^40^**	Advanced UC	Platinum-based CT	Retrospective pooled analysis	DDR alterations; gene-level analysis	Improved survival excluding ATM; poorer outcomes with ATM	Prognostic and treatment-associated associations
**Miron et al.^44^**	MIBC	Cisplatin-based NAC	Retrospective correlative analysis	ATM, RB1, FANCC	Improved 5-year OS and DSS	Treatment-associated association
**SWOG S1314^45^**	MIBC	GC vs ddMVAC	Randomized phase II; correlative analysis	ATM, RB1, FANCC, ERCC2	Absence of alterations had high NPV for pT0	Biomarker association; predictive value not formally demonstrated
**Van Allen et al.^46^**	MIBC	Cisplatin-based NAC	Retrospective genomic analysis	ERCC2	ERCC2 mutations enriched among pathological responders	Treatment-associated association
**Liu et al.^47^**	MIBC	Cisplatin-based NAC	Independent retrospective validation	ERCC2	Association with pathological response replicated	Validated treatment-associated association
**RETAIN-1^48^**	MIBC	NAC → response-adapted surveillance	Prospective single-arm phase II	ATM, ERCC2, FANCC, RB1	Bladder preservation feasible; non-inferiority not met	Clinical feasibility; no formal predictive validation
**Immune checkpoint inhibitors**						
**Teo et al.^36^**	Advanced UC	Anti-PD-1/PD-L1	Retrospective cohort	DDR alterations by pathogenicity	Higher response rates, especially with deleterious alterations	Treatment-associated association
**Joshi et al.^43^**	Advanced UC	Anti-PD-1/PD-L1	Retrospective cohort	DDR alterations	Numerically higher ORR and improved outcomes	Exploratory treatment-associated association
**PURE-01^51^**	MIBC	Neoadjuvant pembrolizumab	Prospective single-arm phase II	Deleterious DDR and/or RB1 alterations	Higher pT0 rates	Exploratory biomarker association
**RETAIN-2^52^**	MIBC	AMVAC + nivolumab → response-adapted surveillance	Prospective single-arm phase II	ATM, ERCC2, or RB1 alterations	Feasibility of biomarker- and response-adapted bladder preservation	Clinical feasibility; predictive value not established
**PARP inhibitor strategies**						
**ATLANTIS^53^**	mUC maintenance	Rucaparib vs placebo after platinum CT	Randomized, double-blind, biomarker-selected phase II	DNA repair deficiency biomarker	PFS signal favoring rucaparib	Activity in selected population; predictive value not formally established
**Meet-URO12^54^**	Advanced UC maintenance	Niraparib vs best supportive care	Randomized phase II	Exploratory HRR subgroup	No PFS benefit overall or in small HRR subgroup	No demonstrated predictive value
**ATLAS^55^**	Previously treated advanced UC	Rucaparib	Open-label single-arm phase II	HRD status	No confirmed responses; short PFS irrespective of HRD	No evidence of clinically relevant activity
**Doroshow et al.^56^**	mUC with DDR alterations	Olaparib	Single-arm phase II	Selected DDR alterations	No objective responses; short PFS	Limited activity in a molecularly selected population
**BAYOU^60^**	Untreated, platinum-ineligible mUC	Durvalumab + olaparib vs durvalumab + placebo	Randomized, double-blind phase II	Exploratory HRR subgroup	Negative overall; PFS signal in HRR-altered tumors	Exploratory predictive signal requiring validation
**BISCAY^61^**	Pretreated advanced UC	Durvalumab + olaparib vs durvalumab	Adaptive biomarker-directed phase Ib platform	HRR alterations	No clear benefit from adding olaparib	No demonstrated predictive value
**NEODURVARIB^62^**	MIBC	Neoadjuvant durvalumab + olaparib	Prospective single-arm phase II	Exploratory DDR analysis	High pathological response; no association with DDR status	Treatment activity not linked to DDR status

Abbreviations: AMVAC, accelerated methotrexate, vinblastine, doxorubicin, and cisplatin; CT, chemotherapy; DDR, DNA damage response; DSS, disease-specific survival; HRD, homologous recombination deficiency; HRR, homologous recombination repair; ICI, immune checkpoint inhibitor; MIBC, muscle-invasive bladder cancer; mUC, metastatic urothelial carcinoma; NAC, neoadjuvant chemotherapy; NPV, negative predictive value; ORR, objective response rate; OS, overall survival; PFS, progression-free survival.

Several studies have attempted to establish a more robust relationship between DDR alterations and response to systemic therapies, with heterogeneous results. Mutations in the HR pathway have been consistently associated with response to PARPi across multiple solid tumors,^67–71^ but in urothelial carcinoma, results are variable: some studies report clinical efficacy in patients with DDR alterations, while others do not. This heterogeneity may be due to a lack of consensus regarding what constitutes a DDR alteration.

In ovarian cancer, patients with high homologous recombination deficiency (HRD) mutational signatures but without classical BRCA1/2 mutations also benefited from PARPi therapy,^72^ suggesting that HR pathway dysfunction can exist without a detectable mutation. In bladder cancer, mutational signatures suggestive of HRD have been identified, such as promoter methylation of RBBP8, which encodes CtIP, a key protein in HR regulation.^38^

Similarly, not all DDR mutations result in functional pathway impairment. Missense mutations, which involve a single amino acid change, can leave the protein partially functional rather than completely inactivated.^73^ Computational systems that use algorithms to predict the functional impact of a gene alteration on the protein may classify mutations as deleterious when they are not clinically relevant and vice versa.^74^^,^^75^

To improve comparability across studies, a harmonized definition of DDR alterations should integrate variant pathogenicity, functional evidence, and pathway-level genomic features rather than relying solely on mutation presence or number. Pathogenic and likely pathogenic alterations according to standardized variant-classification frameworks should be distinguished from variants of uncertain significance, which should not be considered functionally deleterious without supporting evidence. Whenever available, evidence of functional impairment, including biallelic inactivation, loss of heterozygosity, loss of protein expression, or experimentally demonstrated loss of function, should be incorporated. At the pathway level, mutational signatures associated with homologous recombination deficiency, such as SBS3, and genomic scar metrics integrating loss of heterozygosity, telomeric allelic imbalance, and large-scale state transitions may provide a more comprehensive assessment of functional DDR deficiency than mutation counts alone.^30^^,^^38^ Future studies should include a standardized core panel of clinically relevant genes implicated in urothelial carcinoma, including ATM, ERCC2, FANCC, RB1, BRCA1, BRCA2, and PALB2, while allowing broader exploratory panels. Such a multidimensional approach may improve cross-study comparability and facilitate prospective validation of DDR biomarkers.

Even clearly deleterious mutations (BRCA1/2, ATM, CHEK2, PALB2, etc) may have variable effects on treatment sensitivity depending on disease natural history. For instance, an ATM mutation may initially confer chemosensitivity, but in surviving cells, it has been associated with epithelial-to-mesenchymal transition (EMT), increasing invasive capacity.^76^ Jiang et al described ATM as a binary switch determining treatment response according to p53 status: ATM suppression confers chemosensitivity in p53-deficient cells but protects p53-functional cells from chemotherapy.^77^

These findings may partly explain the discordant associations between ATM and other DDR gene alterations and treatment outcomes. Determining the predictive value of each individual gene within DDR pathways is highly complex due to statistical power limitations, necessitating the evaluation of gene panels collectively, with all the inherent limitations this entails.

Nevertheless, DDR alterations remain promising candidate biomarkers that may help identify treatment-associated vulnerabilities to platinum-based chemotherapy, ICIs, and PARP inhibitors. Their use to guide therapeutic sequencing remains investigational and requires prospective validation.

The Atlantis trial showed a PFS benefit in favor of rucaparib (35.3 weeks vs 15.1 weeks; HR, 0.53) in patients with mUC and DDR alterations who had not progressed after platinum-based treatment,^53^ whereas the ATLAS study did not demonstrate clinical benefit in the DDR-mutated (DDRm) subgroup.^55^ This discrepancy may be partly explained by the ATLAS recruiting a pretreated population. The use of PARPi as maintenance aims to consolidate the response achieved with CT, rather than rescue patients in progression who require a rapid response, following a strategy analogous to the JAVELIN Bladder 100 study.^78^ In this regard, although the Meet-URO12 did not show benefit even with a design similar to that of Atlantis (patients who had not progressed on platinum), the proportion of DDR patients was very low (*n* = 6), which may limit the interpretation of the results.^54^ This was due to the early termination of the study following the approval of avelumab maintenance, which rendered the control arm (best supportive care) suboptimal.

The Bayou study demonstrated a significant benefit with the combination of durvalumab + olaparib in DDRm patients,^60^ whereas the BISCAY trial in pretreated patients was negative.^61^ Again, these differences may be explained by the patient profile: more frail populations or those with worse baseline prognosis tend to have poorer outcomes, which can mask efficacy signals.

The incorporation of the EV + P combination in the first-line setting has substantially reshaped the therapeutic landscape of mUC,^7^ potentially limiting the applicability of the classical paradigm of maintenance treatment following platinum-based CT. Nevertheless, the toxicity associated with this combination is not negligible^7^ and may condition the feasibility of prolonged treatment, even in patients who achieve a sustained response.

Analogous to the JAVELIN Bladder 100 trial, which established avelumab as maintenance therapy after 4-6 cycles of CT and enabled the identification of a subgroup of long-term survivors in bladder cancer,^78^ the lack of a clearly defined maintenance strategy following EV + P raises the possibility that selected patient subgroups may continue to benefit from a CT-based approach followed by avelumab maintenance in the first-line setting. In this context, the identification of predictive biomarkers of benefit from CT becomes particularly relevant. The sequencing of immunotherapy after EV + P has not demonstrated clinically meaningful efficacy,^79–81^ and therefore avelumab maintenance does not currently represent a standardized option in this setting.

On the other hand, the optimal therapeutic sequence for patients who progress after EV + P remains unclear. In some cases, residual neuropathy associated with EV may limit the subsequent use of platinum-based CT. In this context, alterations in DDR genes have shown relatively consistent associations with favorable outcomes following platinum-based CT,^40^^,^^41^^,^^45–47^ although treatment-specific predictive value has not been formally demonstrated. These findings support their further evaluation as candidate biomarkers for identifying patients who may derive greater clinical benefit from platinum-based strategies and for optimizing the risk-benefit balance of treatment. Prospective validation of this approach is currently being evaluated in the SELECTIO-UC study.^49^ Furthermore, in patients with DDR-altered tumors who receive platinum-based CT after progression, the role of PARPi as a subsequent therapeutic strategy could be considered as a hypothesis to be explored in future clinical trials, particularly in a context in which avelumab maintenance would no longer be an option.

Given the growing interest in exploiting the susceptibility induced by DDR alterations, new therapeutic strategies targeting key proteins in the regulation of these pathways are emerging.

Cell line-based models have shown that in urothelial cells with functional DDR (which depend on these pathways to acquire cisplatin resistance), Chk1 inhibition can restore platinum sensitivity.^82^

WEE1 acts as a key regulator of DDR checkpoints by inhibiting CDK1/2 and allowing DNA repair; thus, its inhibition forces DDR-deficient cells into mitosis with unrepaired damage, inducing cell death. WEE1 inhibition has been explored as a potential therapeutic target in combination with CT in patients with mUC and DDRm.^83–85^

Similarly, ATR or CHK1 inhibition has shown synergy in preclinical models of DDRm urothelial carcinoma.^86^^,^^87^

This range of strategies, still under development, reinforces the potential of DDR-targeted therapies as an emerging cornerstone in the treatment of urothelial carcinoma.

**Figure 1.**
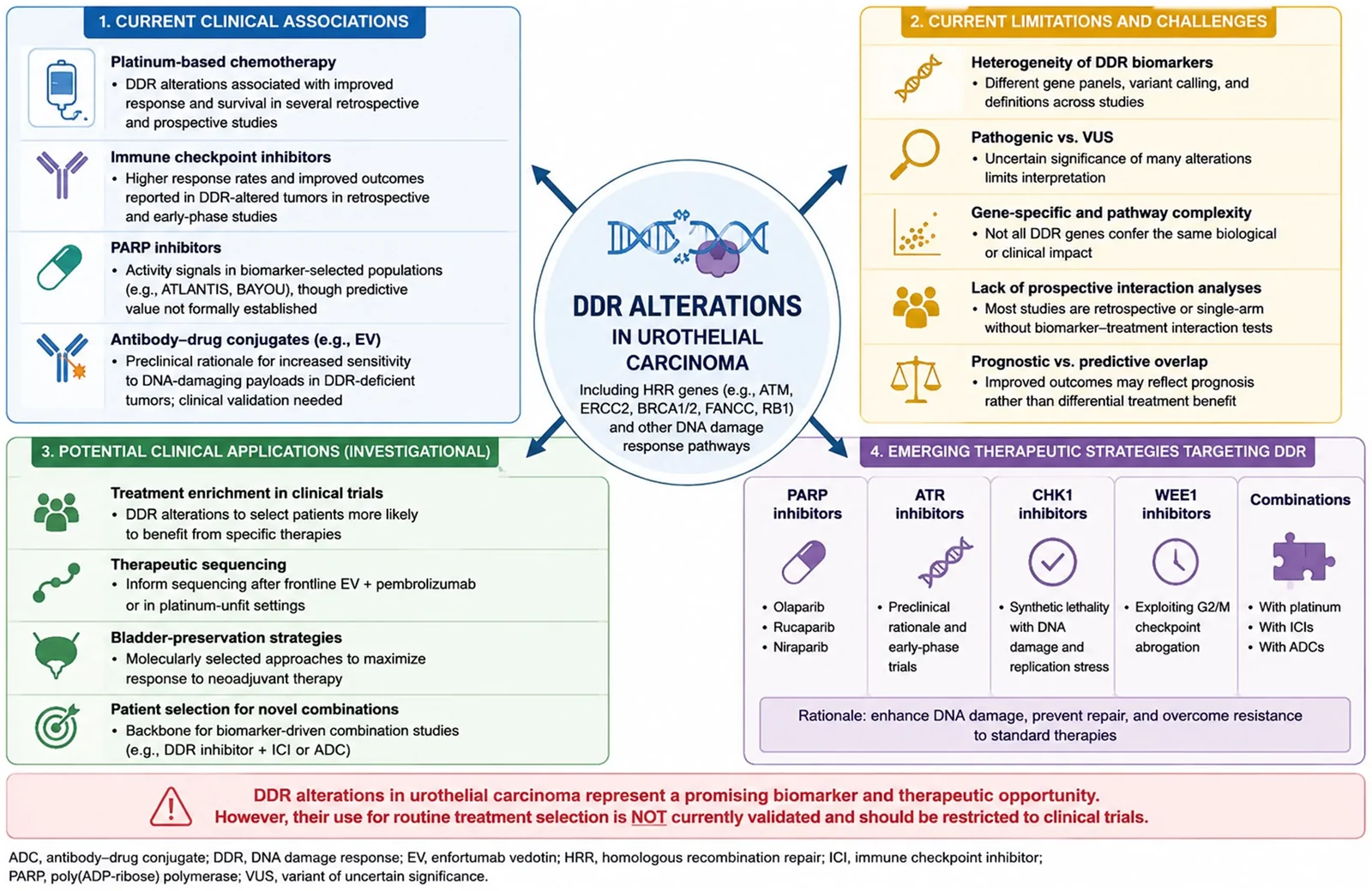
Conceptual framework for the clinical relevance and therapeutic targeting of DDR alterations in urothelial carcinoma. The figure was generated with the assistance of OpenAI’s image-generation tool and subsequently reviewed and edited by the authors for scientific accuracy and clarity.

This framework is presented in Figure 1.

## Conclusions

Alterations in DDR pathways have gained increasing interest in urothelial carcinoma as candidate prognostic and treatment-associated biomarkers. Their assessment may ultimately help refine therapeutic sequencing and support more individualized treatment strategies; however, prospective randomized validation with formal biomarker-treatment interaction analyses is required before DDR biomarkers can be incorporated into routine treatment selection.

## Data Availability

No new datasets were generated or analyzed in support of this research.
